# A New Superhard *sp*^3^-Hybridized Carbon Allotrope with Ultrawide Direct Band Gap: *Ibca*-C_64_

**DOI:** 10.3390/ma18184316

**Published:** 2025-09-15

**Authors:** Xinyu Wang, Qun Wei, Jing Luo, Meiguang Zhang, Bing Wei

**Affiliations:** 1 School of Physics, Xidian University, Xi’an 710071, China; 2 College of Physics and Optoelectronic Technology, Baoji University of Arts and Sciences, Baoji 721016, China

**Keywords:** carbon, superhard materials, mechanical and electronic properties

## Abstract

A novel all-*sp*^3^-hybridized superhard carbon allotrope, *Ibca*-C_64_, is proposed based on first-principles calculations combined with the RG^2^ (space group and graph theory) structure search method. A systematic investigation of its stability, mechanical properties, and electronic structure is performed. The results indicate that the energy difference between *Ibca*-C_64_ and diamond is only 0.295 eV/atom, suggesting its metastability. Detailed analysis of its elastic constants and phonon spectrum confirms both mechanical and dynamical stability. The *Ibca*-C_64_ structure demonstrates exceptional mechanical performance, with a Vickers hardness of 83.9 GPa. Furthermore, it possesses a wide direct band gap of 5.58 eV, indicating that *Ibca*-C_64_ is a superhard semiconductor material with outstanding mechanical properties.

## 1. Introduction

Carbon, owing to its unique bonding characteristics, can form a diverse range of allotropes—from the well-known diamond and graphite to fullerenes, carbon nanotubes, and graphene. The discovery and investigation of these allotropes have substantially advanced the field of materials science [1,2,3,4,5,6,7,8]. Among them, diamond is notable for its exceptional hardness and high thermal conductivity, making it widely used in cutting, drilling, grinding, and aerospace applications. However, the high production cost and limited environmental adaptability of diamond restrict its use in specific scenarios such as high-temperature machining and the cutting of iron-based materials. Consequently, researchers have sought new carbon allotropes that combine superhardness with chemical and thermal stability, and favorable electronic properties.

In recent years, theoretical calculations have played an increasingly important role in the design of carbon-based materials. Using density functional theory (DFT) and crystal structure prediction algorithms, numerous structurally novel carbon allotropes have been proposed, including *R*16 carbon [9], *T*-C8 [10], *hP*-C17 [11], *hP*-C18 [12], *O*16 carbon [13], *T*-C64 [14], Orth-C10, Orth-C10′ [15], and *T*5-carbon [16]. Many of these exhibit superhard characteristics and desirable physical properties. For instance, Lv et al. [17] reported *tri*-C18, a superhard carbon allotrope with a wide indirect band gap of 6.32 eV. Its simulated X-ray diffraction pattern matches previously unexplained peaks found in chimney soot, suggesting its potential existence in that environment. Hussain et al. [18] proposed an all-*sp*^3^-hybridized superhard carbon material, *m*-C16, composed of five-, six-, and seven-membered rings. This phase demonstrates mechanical properties approaching those of diamond, along with favorable ultraviolet–visible absorption. Additionally, Ju et al. [19] introduced *T*20 carbon, a superhard carbon allotrope predicted via first-principles calculations to have a Vickers hardness of approximately 83.5 GPa and an indirect band gap of about 5.80 eV. Liu et al. [20] proposed four all-*sp*^3^ hybridized superhard carbon allotropes in the *P*4_2_*/mmc* space group (*P*4_2_*/mmc* C_32_-I, *P*4_2_*/mmc* C_32_-II, *P*4_2_*/mmc* C_36_, *P*4_2_*/mmc* C_40_). These structures feature various ring motifs and exhibit mechanical, dynamical, and thermal stability at 500 K, along with low relative enthalpies. Mukherjee et al. [21] proposed an *sp*^2^–*sp*^3^ hybridized superhard carbon allotrope, poC_36_, which is an indirect-gap semiconductor at ambient pressure and develops 1D conductivity at around 11 GPa. Despite these advances, most *sp*^3^-hybridized superhard carbon allotropes possess indirect band gaps, limiting their applicability in optoelectronic and deep-ultraviolet photoelectric devices. Thus, the search for new carbon materials that simultaneously exhibit superhardness and a wide direct band gap remains both valuable and necessary.

In view of the limitations of most of the aforementioned superhard carbon materials, we predict a novel all-*sp*^3^ hybridized superhard carbon material, *Ibca*-C_64_, with a wide direct band gap, using the space group and graph theory (RG^2^) method [22]. This structure overcomes the common drawback of indirect band gaps in existing superhard carbons. To further explore the properties of *Ibca*-C_64_, we performed first-principles calculations to investigate its stability, mechanical performance, and electronic characteristics, and carried out a comparative analysis against other superhard carbon allotropes reported in the literature. This discovery expands the known structural diversity of carbon allotropes and provides a theoretical foundation for the development of future high-performance carbon materials. Moreover, our results also provide a promising candidate for the development of practical materials that combine superhard properties with excellent optoelectronic performance, suggesting potential applications in cutting tools and in wear-resistant electronic devices operable in the deep ultraviolet (UV) region. It is important to clarify that the current study remains purely theoretical, focusing on the prediction and computational characterization of the *Ibca*-C_64_ structure without experimental synthesis or validation. Nevertheless, our first-principles calculations have yielded clear and unambiguous results regarding structural stability, mechanical properties, and electronic structure. These results (e.g., calculated elastic constants, hardness, and wide direct band gap) provide specific targets and guidance for future experimental work.

## 2. Computational Details

The RG^2^ method has been widely used to construct crystal structures with specific topological features and has proven effective in generating carbon allotropes [23,24]. Some initial parameters were selected to constrain the predicted structures to orthorhombic crystals exhibiting *sp*^3^ hybridization. The unit cell was set to contain 64 atoms, constrained to the orthorhombic space group, and each carbon atom was bonded to four others to ensure *sp*^3^ hybridization. Subsequent structural optimizations and property calculations were performed within the framework of density functional theory (DFT) [25] using the Vienna Ab initio Simulation Package (VASP 5.4.4) [26]. Based on previous investigations of carbon allotropes, the projector augmented wave (PAW) method [27] was employed in conjunction with the Perdew–Burke–Ernzerhof (PBE) [16,17,18,19,28] exchange–correlation functional under the generalized gradient approximation (GGA) [29]. A cutoff energy of 900 eV was used for plane-wave expansion, and a Monkhorst–Pack [30] *k*-point grid with a spacing of 2π × 0.02 Å^−1^ was applied to ensure total energy convergence within 1 × 10^−5^ eV/atom. A 2 × 1 × 1 supercell with 128 atoms were used to calculate the phonon dispersions of *Ibca*-C_64_. The electronic band structures were calculated using the Heyd–Scuseria–Ernzerhof (HSE06) hybrid functional [31]. Phonon spectra were obtained using the PHONOPY (version 2.9.0) package [32].

## 3. Results and Discussion

### 3.1. Crystal Structure and Structure Stability

**Figure 1:**
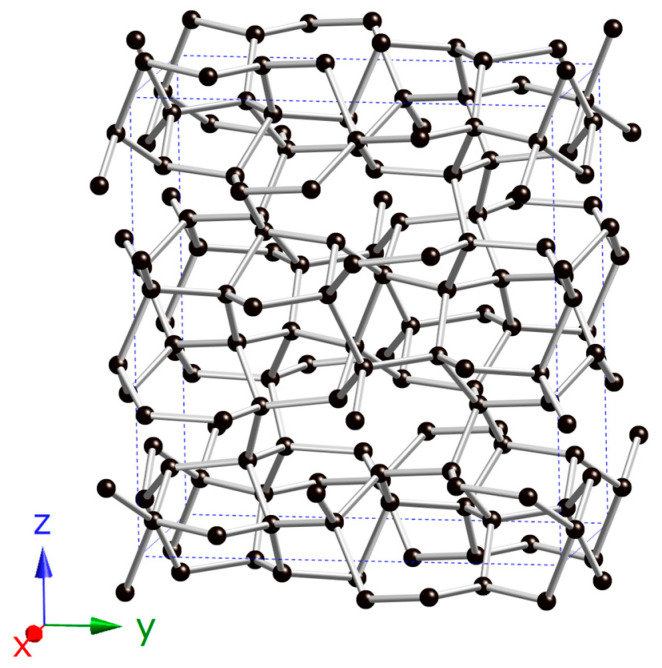
Crystal structures of *Ibca*-C_64_.

**Figure 2:**
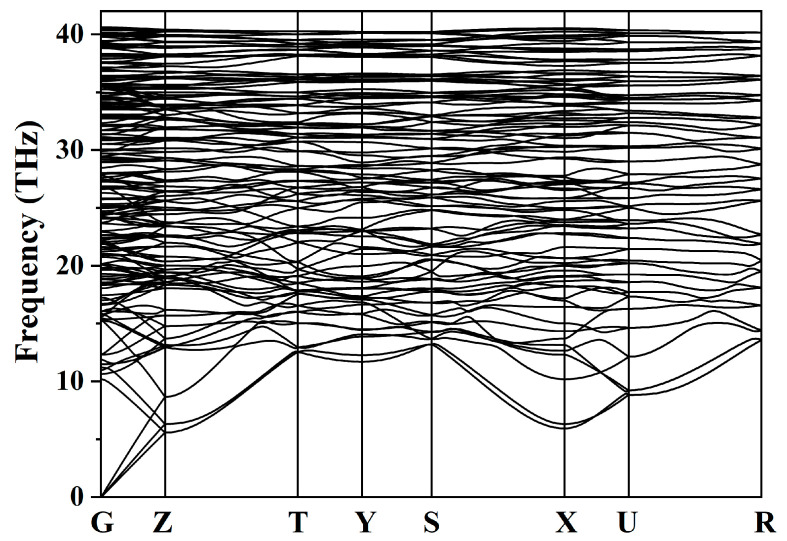
Phonon spectra of *Ibca*-C_64_.

A novel carbon allotrope, *Ibca*-C_64_, was predicted using the RG^2^ method, and its topological structure was analyzed with TopCryst [33]. The analysis revealed that *Ibca*-C_64_ exhibits a 4^4^-c net topology, which is not recorded in the Samara Carbon Allotrope Database (SACADA) [34]. Furthermore, we compared it with other C_64_ allotropes reported in the literature [14,28,35,36,37] and found that *Ibca*-C_64_ exhibits a topological structure that is significantly different from previously proposed C_64_ allotropes, indicating that it represents a previously unknown carbon atomic network. Its unit cell contains 64 atoms and is shown in Figure 1. The structure is fully *sp*^3^-hybridized. Four inequivalent carbon atoms occupy Wyckoff positions C1 16f (0.6458, 0.7979, 0.0624), C2 16f (0.5247, 0.4619, 0.9190), C3 16f (0.8261, 0.7250, 0.1836), and C4 16f (0.7400, 0.5478, 0.8304). Table 1 lists the crystal structure parameters and density of *Ibca*-C_64_ in comparison with other carbon allotropes. The density of *Ibca*-C_64_ is 3.465 g/cm^3^, which is significantly higher than that of the two other C_64_ allotropes considered, and only 0.08 g/cm^3^ lower than that of diamond. This high density suggests potential for superhardness. The energy difference between *Ibca*-C_64_ and diamond is 0.295 eV/atom, which is lower than that of most carbon allotropes in SACADA, indicating favorable thermodynamic metastability and suggesting a high likelihood of synthesizability. In the orthorhombic system, there are nine independent elastic constants: *C*_11_, *C*_12_, *C*_13_, *C*_22_, *C*_23_, *C*_33_, *C*_44_, *C*_55_, and *C*_66_. These values were calculated and listed in Table 2. The mechanical stability according to the Born stability criteria are [38] $C_{11}>0$, $C_{22}>0$, $C_{33}>0$, $C_{44}>0,$ $C_{55}>0$, $C_{66}>0$, $C_{11}C_{22}>C_{12}^{2},$ and $2C_{12}C_{13}C_{23}+C_{11}C_{22}C_{33}-C_{11}C_{23}^{2}-C_{22}C_{13}^{2}-C_{33}C_{12}^{2}>0.$ The results show that *Ibca*-C_64_ satisfies the Born criteria, confirming its mechanical stability. Notably, although the *C*_11_ and *C*_22_ values are slightly lower than those of *Imma*-carbon, they are higher than those of diamond, *I*4_1_/*amd*-C_64_, *T*-C_64_, and *M*-carbon, indicating that *Ibca*-C_64_ possesses superior compressive strength along the *x*- and *y*-axes under linear compression. The phonon spectrum of *Ibca*-C_64_ is shown in Figure 2. All phonon modes exhibit positive frequencies across the entire Brillouin zone, confirming the dynamical stability of the structure.

### 3.2. Mechanical Properties

To examine the mechanical properties of Ibca-C64, the bulk modulus (B) and shear modulus (G) were derived from the elastic constants via the Voigt–Reuss–Hill approximation [39], a widely utilized approach for computing elastic constants and moduli [40,41]. The results are presented in Table 2. *Ibca*-C_64_ exhibits a bulk modulus of 409 GPa and a shear modulus of 473 GPa, both relatively high and comparable to those of *M*-carbon and *Imma*-carbon, though slightly lower than those of diamond. These values indicate strong resistance to both compression and shear. The calculated *B*/*G* ratio of *Ibca*-C_64_ is 0.86, which is close to that of diamond (0.83), indicating a brittle mechanical nature [42]. According to Chen’s model [43], the Vickers hardness of *Ibca*-C_64_ is estimated to be 83.9 GPa (Table 2). This value is only 10 GPa lower than that of diamond and exceeds those of *I*4_1_/*amd*-C_64_, *T*-C_64_, *M*-carbon, and *Imma*-carbon, confirming that *Ibca*-C_64_ is a superhard material with considerable potential for applications requiring extreme hardness. Elastic anisotropy is a critical factor in practical applications. The mechanical response of *Ibca*-C_64_ along various crystallographic directions was examined in order to characterize this property. For orthorhombic crystals, the directional dependence of Young’s modulus (*E*) and torsional shear modulus (*G*_t_) is given by

(1) $$\begin{array}{c} E^{-1}=R_{1}^{4}s_{11}+R_{2}^{4}s_{22}+R_{3}^{4}s_{33}+R_{2}^{2}{R_{3}^{2}(2s}_{23}+s_{44})\\ {+R}_{1}^{2}{R_{3}^{2}(2s}_{13}+s_{55}){+R}_{1}^{2}{R_{2}^{2}(2s}_{12}+s_{66}) \end{array}$$

and

(2) $$\begin{array}{l} G_{t}^{-1}=2s_{11}R_{1}^{2}\left(1-R_{1}^{2}\right)+2s_{22}R_{2}^{2}\left(1-R_{2}^{2}\right)+2s_{33}R_{3}^{2}\left(1-R_{3}^{2}\right)\\ -4s_{12}R_{1}^{2}R_{2}^{2}-4s_{13}R_{1}^{2}R_{3}^{2}-4s_{23}R_{2}^{2}R_{3}^{2}\\ +\frac{1}{2}s_{44}\left(1-R_{1}^{2}-4R_{2}^{2}R_{3}^{2}\right)+\frac{1}{2}s_{55}\left(1-R_{2}^{2}-4R_{1}^{2}R_{3}^{2}\right)\\ +\frac{1}{2}s_{66}(1-R_{3}^{2}-4R_{1}^{2}R_{2}^{2}) \end{array}$$

**Figure 3:**
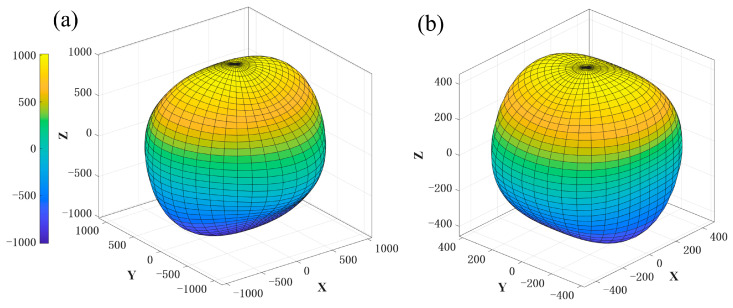
Directional dependence of (**a**) Young’s modulus and (**b**) torsional shear modulus of *Ibca*-C_64_ (in GPa).

In Equation (1), *R*_1_, *R*_2_, and *R*_3_ represent the direction cosines of a given crystallographic orientation [*hkl*] relative to the coordinate axes *x*_1_, *x*_2_, and *x*_3_, and *s*_ij_ are the elastic compliance constants. In Equation (2), the same directional cosines describe the torsion axis for the corresponding [*hkl*] orientation. The calculated directional dependence of Young’s and torsional shear moduli is illustrated in Figure 3, this figure clearly illustrates the variation in elastic stiffness of the *Ibca*-C_64_ structure across different crystallographic directions. The surfaces depicted in Figure 3 markedly deviate from a sphere, revealing significant elastic anisotropy in the *Ibca*-C_64_ structure. Elastic anisotropy is associated with microcrack formation and lattice deformation, these results can pinpoint the strongest and most deformation-prone directions in the *Ibca*-C_64_ structure, thus guiding its directional design in practical applications to prevent overload along weaker orientations.

**Figure 4:**
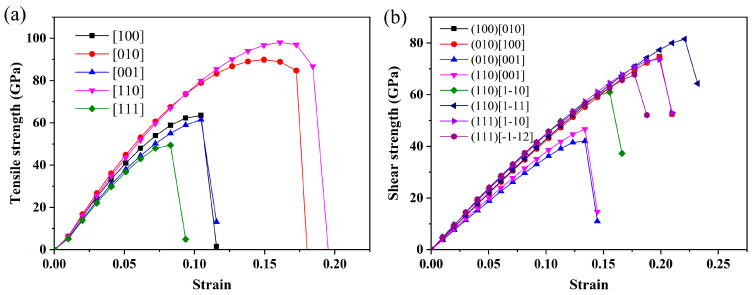
Tensile strength (**a**) and shear strength (**b**) of *Ibca*-C_64_ as functions of strain.

The ideal tensile and shear strengths of *Ibca*-C_64_ were plotted as functions of strain (Figure 4) in order to further explore its mechanical strength. The minimum tensile strength, 49.5 GPa, occurs along the [111] direction, while the maximum tensile strength, 86.8 GPa, is observed along [110]. These results suggest that under uniaxial tension, fracture is most likely to initiate along the (111) plane. Figure 4b shows the directional dependence of shear strength. The (010)[001] orientation exhibits the lowest shear strength, 42.1 GPa—lower than the minimum tensile strength. This indicates that shear-driven deformation is the dominant failure mechanism in *Ibca*-C_64_ under stress.

### 3.3. Electronic Properties

**Figure 5:**
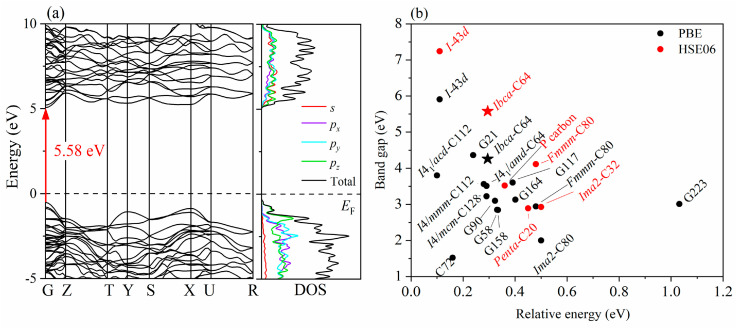
(**a**) Electronic band structure and the density of states of *Ibca*-C_64_ calculated using the HSE06 hybrid functional. The dashed line at 0 eV represents the Fermi energy level. (**b**) Band gaps of *Ibca*-C_64_ and some other known direct band gap carbon allotropes vs. relative energy referenced to diamond (The star symbol in the figure denotes the *Ibca*-C_64_ phase).

The band structure of *Ibca*-C_64_ was calculated using the HSE06 hybrid functional to analyze its electronic properties, as shown in Figure 5a. The results clearly indicate that *Ibca*-C_64_ possesses a wide direct band gap of 5.58 eV. For comparison, the band gap of *Ibca*-C_64_ and some other known carbon allotropes with a direct band gap are shown in Figure 5b. It can be seen that *I*-43*d*-carbon has the widest direct band gap, whereas that of *Ibca*-C_64_ is the third widest. This confirms its identity as a superhard semiconductor with a wide direct band gap. Furthermore, Figure 5a shows that near the Fermi level, the energy is mainly contributed by the C-*p* orbitals.

### 3.4. Simulated X-Ray Diffraction (XRD) Pattern

**Figure 6:**
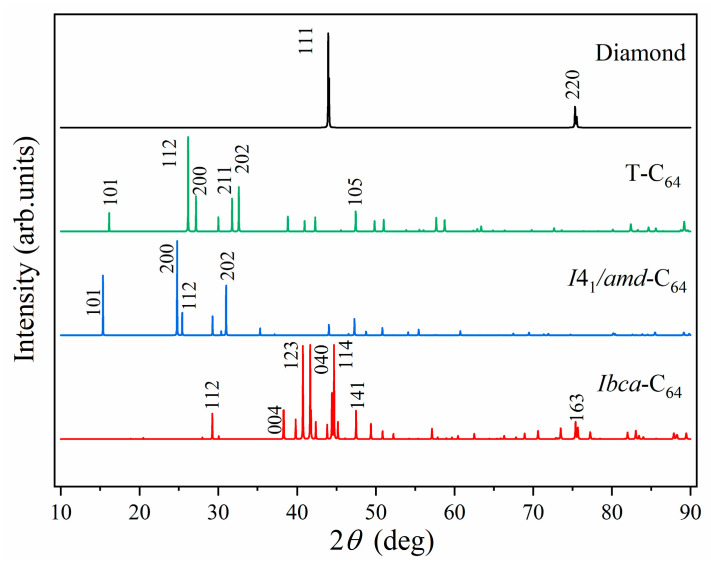
Simulated X-ray diffraction (XRD) pattern for *Ibca*-C_64_, *I*4_1_/*amd*-C_64_, *T*-C_64_, and diamond. The X-ray wavelength is 1.5406 Å with a copper source.

Finally, to further provide more detailed structural information for the experimental identification of *Ibca*-C_64_, we simulated its X-ray diffraction (XRD) pattern and compared it with those of *I*4_1_/*amd*-C_64_, *T*-C_64_, and diamond (Figure 6). The XRD profile of *Ibca*-C_64_ exhibits seven principal peaks at 29.26°, 38.30°, 40.73°, 41.65°, 44.68°, 47.48°, and 75.39°. The strongest reflection occurs at 44.68°, corresponding to the (114) crystallographic plane. Additionally, within the 40–45° range, two other pronounced peaks appear, corresponding to the (123) and (040) planes. These diffraction peaks differ markedly from those of the other C_64_ allotropes. Notably, the peaks near 44.68° and 75.39° are very close to diamond’s reflections. These characteristic peaks serve as critical structural fingerprints for phase identification and characterization of *Ibca*-C_64_, and we anticipate that they will assist in its experimental detection.

### 3.5. Discussion

Compared with other recently proposed *sp*^3^ carbon allotropes, *Ibca*-C_64_ offers a distinctive balance of properties. For example, allotropes such as *m*-C16 [18] and T20 [19] carbon achieve near-diamond hardness (≈83–90 GPa) but possess indirect band gaps, limiting their applicability in optoelectronic and deep-ultraviolet photoelectric devices. By contrast, *Ibca*-C_64_ combines comparable superhardness with a wide direct band gap, making it particularly attractive for deep-ultraviolet optoelectronic applications and industrial uses requiring extreme hardness. The calculations presented above confirm this conclusion. According to Chen’s empirical model, the Vickers hardness of *Ibca*-C_64_ is estimated to be 83.9 GPa. Although this value is approximately 10 GPa lower than that of diamond, it surpasses the hardness reported for other C_64_ allotropes and numerous known carbon phases, thereby supporting the classification of *Ibca*-C_64_ as a superhard material. Notably, electronic band structure calculations indicate that *Ibca*-C_64_ possesses a wide direct band gap of 5.58 eV. For comparison, the band gap of *Ibca*-C_64_ was compared with those of several other direct band gap carbon allotropes reported in the literature. Among the compared phases, *I*-43*d*-carbon exhibits the widest direct band gap, whereas that of *Ibca*-C_64_ is the third widest. The results indicate that *Ibca*-C_64_ is a superhard semiconductor possessing a wide direct band gap.

In addition, compared with the two other C_64_ allotropes reported in the literature [14,28], *Ibca*-C_64_ exhibits significantly higher Vickers hardness and a larger band gap, indicating superior performance. Moreover, its relatively low formation energy (~0.295 eV/atom above diamond) suggests that appropriate high-pressure or plasma-based synthesis routes could access this metastable phase. Overall, the co-occurrence of superhardness and a wide direct band gap in *Ibca*-C_64_ indicates significant potential for next-generation deep-ultraviolet devices and abrasive tools, enriching the family of carbon allotropes.

## 4. Conclusions

To identify a novel carbon allotrope exhibiting both superhardness and a wide direct band gap, a novel carbon allotrope, *Ibca*-C_64_, was predicted using the RG^2^ method, and its structural, mechanical, and electronic properties were investigated through first-principles calculations. *Ibca*-C_64_ exhibits a previously unknown 4^4^-c net topology and an all *sp*^3^-hybridized orthorhombic cell containing 64 atoms. Its calculated density (3.465 g/cm^3^) is extremely high (only 0.08 g/cm^3^ below diamond), and its total energy is 0.295 eV/atom above that of diamond, indicating both potential superhardness and feasible metastability. All elastic constants satisfy the Born stability criteria, and phonon calculations show no imaginary modes, confirming mechanical and dynamical stability.

The directional dependence of Young’s modulus and torsional shear modulus confirms that *Ibca*-C_64_ exhibits significant elastic anisotropy. According to Chen’s empirical model, the Vickers hardness of *Ibca*-C_64_ is predicted to be 83.9 GPa, indicating that *Ibca*-C_64_ qualifies as a superhard material. Moreover, electronic band structure calculations indicate that *Ibca*-C_64_ has a wide direct band gap of 5.58 eV. For comparison, the band gap of *Ibca*-C_64_ was compared with those of several other direct band gap carbon allotropes reported in the literature. The results indicate that *Ibca*-C_64_ is a superhard semiconductor possessing a wide direct band gap. The simulated XRD pattern shows seven dominant diffraction peaks that differ markedly from those of other C_64_ forms and closely align with major diamond reflections, providing distinctive fingerprints for potential experimental identification. These characteristics make *Ibca*-C_64_ a promising candidate for wear-resistant electronic devices designed to operate in the deep ultraviolet region and for cutting tools. Moreover, this study enriches the family of all-*sp*^3^ hybridized carbon allotropes and provides a solid theoretical foundation for experimental synthesis.

## Figures and Tables

**Table 1:** Calculated Lattice parameters and density of *Ibca*-C_64_, *I*4_1_/*amd*-C_64_, *T*-C_64_, and diamond.

Phases	*a* (Å)	*b* (Å)	*c* (Å)	*ρ* (g/cm^3^)
*Ibca*-C_64_	4.525	8.667	9.394	3.465
*I*4_1_/*amd*-C_64_ [28]	7.180		9.665	2.562
*T*-C_64_ [14]	6.557		10.012	2.966
Diamond [14]	3.566			3.517

**Table 2:** Calculated bulk modulus (*B*, GPa), shear modulus (*G*, GPa), Young’s modulus (*E*, GPa), *B*/*G* ratio, Poisson’s ratio (*ν*), and Vickers hardness (*H*_v_, GPa) for *Ibca*-C_64_ and other carbon allotropes.

	*Ibca*-C_64_	*I*4_1_/*amd*-C_64_ [28]	*T*-C_64_ [14]	*M*-Carbon [28]	*Imma*-Carbon [28]	Diamond [14]
*C*_11_	1079	598	525	929	1126	1053
*C*_22_	1149			1087	1105	
*C*_33_	1025	677	926	1044	1196	
*C*_44_	392	254	369	521	446	563
*C*_55_	525			452	526	
*C*_66_	450	107	369	389	377	
*C*_12_	79	43	106	49	86	120
*C*_13_	119	108	91	156	121	
*C*_23_	18			87	61	
*C*_15_				63		
*C*_25_				−28		
*C*_35_				26		
*C*_46_				−8		
*B*	409	264	276	404	408	431
*G*	473	217	324	454	476	522
*E*	1024	510	699	991	1049	1116
*υ*	0.080	0.178	0.077	0.090	0.102	0.070
*B*/*G*	0.86	1.22	0.85			0.83
*H*_v_	83.9	33.9	68.2	81.6	79.6	93.9

## Data Availability

The original contributions presented in the study are included in the article. Further inquiries can be directed to the corresponding authors.
